# Clinical manifestations of severe enterovirus 71 infection and early assessment in a Southern China population

**DOI:** 10.1186/s12879-017-2228-9

**Published:** 2017-02-17

**Authors:** Si-da Yang, Pei-qing Li, Yi-min Li, Wei Li, Wen-ying Lai, Cui-ping Zhu, Jian-ping Tao, Li Deng, Hong-sheng Liu, Wen-cheng Ma, Jia-ming Lu, Yan Hong, Yu-ting Liang, Jun Shen, Dan-dan Hu, Yuan-yuan Gao, Yi Zhou, Min-xiong Situ, Yan-ling Chen

**Affiliations:** 1 Guangzhou Women and Children’s Medical Center, Guangzhou Medical University, Guangzhou, 510623 China; 2 Respiratory Research Institute, the First Affiliated Hospital of Guangzhou Medical University, Guangzhou, 510120 China; 3 Dongguan Taiping People’s Hospital, Dongguan, 523905 China; 4 Zhongshan People’s Hospital, Zhongshan, 528403 China

**Keywords:** Enterovirus 71, Clinical manifestations, Children, Southern China

## Abstract

**Background:**

Enterovirus 71 (EV-A71) shows a potential of rapid death, but the natural history of the infection is poorly known. This study aimed to examine the natural history of EV-A71 infection.

**Methods:**

This was a prospective longitudinal observational study performed between January 1^st^ and October 31^st^, 2012, at three hospitals in Guangdong, China. Subjects with positive EV-A71 RNA laboratory test results were included. Disease progression was documented with MRI, autopsies, and follow-up. Symptoms/signs with potential association with risk of death were analyzed.

**Results:**

Among the 288 patients, neurologic symptoms and signs were observed (emotional movement disorders, dyskinesia, involuntary movements, autonomic dysfunction, and disturbance of consciousness). Some of them occurred as initial symptoms. Myoclonic jerks/tremors were observed among >50% of the patients; nearly 40% of patients presented fatigue and 25% were with vomiting. Twenty-eight patients (9.7%) presented poor peripheral perfusion within 53.4 ± 26.1 h; 23 patients (8.0%) presented pulmonary edema and/or hemorrhage within 62.9 ± 28.6 h. Seventeen (5.9%) patients were in a coma. Seven (2.4%) patients died within 62.9 ± 28.6 h. Seventy-seven survivors underwent head and spinal cord MRI and 37.7% (29/77) showed abnormalities. Two fatal cases showed neuronal necrosis, softening, perivascular cuffing, colloid, and neuronophagia phenomenon in the brainstem.

**Conclusions:**

Patients with EV-A71 infection showed high complexity of symptoms and onset timing. Death risk may be indicated by autokinetic eyeball, eyeball ataxia, severe coma, respiratory rhythm abnormality, absent pharyngeal reflex, ultrahyperpyrexia, excessive tachycardia, pulmonary edema and/or hemorrhage, and refractory shock and ataxic respiration. Early assessment of these symptoms/signs is important for proper management.

**Electronic supplementary material:**

The online version of this article (doi:10.1186/s12879-017-2228-9) contains supplementary material, which is available to authorized users.

## Background

Recently, the enterovirus 71 (EV-A71) has been considered as the second most significant neurotropic enterovirus, next only to poliovirus [1]. EV-A71 infection may rapidly lead to fatal pulmonary edema [2]. During the 2008 outbreak of hand, foot, and mouth disease (HFMD) in mainland China, EV-A71 was responsible for 45% of mild cases, 80% of severe cases, and 93% of fatal cases [3]. In cases of EV-A71 infection, the median time from symptom onset to death has been reported to be 3.5 days and only 0.5 day between diagnosis and death [3]. During this outbreak, most (93%) of the non-surviving patients were confirmed to be infected with EV-A71 and some of these patients died before laboratory results were back, the median time between diagnosis and death being 0.5 day [3]. Neuroimaging and laboratory testing may not offer a practical assessment in severely affected patients in the general clinical settings since results will be obtained after dramatic outcomes. Hence, diagnosis and evaluation of severity based on clinical observation is of great value. It has been well shown that neurological system involvement plays an important role in the rapid progression to death. However, due to the complexity of symptoms and onset timing, there is a lack of comprehensive knowledge about the association between clinical manifestations and disease progression.

Analysis of the 1998 Taiwan EV-A71 HFMD outbreak showed that complications included encephalitis, pulmonary edema or hemorrhage, aseptic meningitis, myocarditis, and paralysis were associated with EV-A71 infection [4–6]. Analyses of the 1997 and 2000 Malaysia outbreaks revealed similar complications and causes of death among children infected with EV-A71 [7]. Earlier studies also revealed that neurological involvement was frequent in EV-A71 infection [8, 9]. A review of the literature showed that symptoms could be classified into prodromal indicators, vague indicators, dyskinesia, autonomic dysfunction, and disturbance of consciousness [10–12].

Therefore, this prospective observational study was carried out to characterize the various symptoms of EV-A71 infection and to explore the associations between clinical manifestations and their indications for disease severity and progression.

## Methods

### Study design

This was a prospective, longitudinal observation aiming to record the clinical manifestations and onset timing of cases with severe EV-A71 infection. A study board consisting of pediatricians, pediatric neurologists, and HFMD specialists of the National Health and Family Planning Commission of The People’s Republic of China, and experts of the National Clinical Research Center for Respiratory Diseases and of the Institute of Neuroscience of Guangzhou Medical University was organized in 2008. By reviewing case records of HFMD from our experience as well as from previous studies [10–12], 91 symptoms and signs associated with EV-A71 infection were listed and used as observational indicators. Standard forms were created to record the manifestations and timing of progression. All participating pediatricians were specifically trained to use the standard form to record symptoms and signs of suspected cases of EV-A71 infection. The time at the appearance of the first observed symptom or sign among those 91 indicators was defined as the onset of illness. The occurrence and duration of various symptom and signs were recorded in terms of hours. Treatment of the severe cases was made according to the available national guidelines (http://www.gov.cn/gzdt/2008-05/03/content_960347.htm, which is similar to an English version from Hong Kong: http://www.dh.gov.hk/chs/useful/useful_ld/files/ltod20070523.pdf). This study was approved by the Ethics Committees of all three participating hospitals. Written informed consent was obtained from the legal guardians of the patients.

Cases of EV-A71 were observed between January 1^st^ and October 31^st^, 2012 at three hospitals: the Guangzhou Women and Children’s Medical Center Affiliated to Guangzhou Medical University and Sun Yat-sen University, the Dongguan Taiping People’s Hospital, and the Zhongshan People’s Hospital, all of which are major treatment centers of HFMD in the coastal cities of the Guangdong province. The three centers are located in the core area of the Zhujiang Delta, where it is dominated by the monsoon climate from the South Asian Ocean. This region presents a high HFMD incidence. Autopsies were performed at the Forensic Medical Identification Center of Sun Yat-Sen University.

### Participants

Patients <14 years old with suspected EV-A71 infections (patients with HFMD or patients without rash or herpangina but with neurological symptoms) were screened by real-time PCR using a throat swab sample, as previously described [13]. Subjects with positive EV-A71 RNA laboratory test results (ABI 7300 real time fluorescence quantitative PCR; Ct value <34.9 was considered positive) were then included; any patients positive for EV-A71 in combination to any other virus were excluded. The exclusion criteria were: 1) tested positive for any pathogens other than EV-A71 (including any other enterovirus or CoxA16); 2) trauma; 3) intoxication; 4) received any vaccine within 4 weeks (to exclude patients with increased susceptibility to EV-A71 because of immune reaction to the vaccine or confounding symptoms from the vaccine); or 5) other central nervous system (CNS) diseases such as cerebral palsy or intracranial space-occupying lesion. Therefore, 288 patients were included. The legal guardians signed the consent forms. MRI or autopsies were performed in some participants with the permission from the patients or their legal guardians.

### Patient assessment

Patients were triaged in a standard manner, which determined the interval of the clinical assessments. Critical patients were continuously monitored. Emergency patients were assessed at 15-min intervals. Urgent patients were assessed at 15–30 min intervals. There was no non-urgent patients in the present study.

### Magnetic resonance imaging

MRI was performed using a PHILIPS Achieve 1.5 T (Philips, Best, The Netherlands). One senior radiologist and one senior neurologist analyzed the MRI scans.

### Statistical analysis

Descriptive statistics were used to interpret the patterns of the 91 observational factors and the sequences of occurrence. The Student’s *t*-test was used to detect differences in occurrence timing of each symptom. Correlations were assessed using the Pearson’s test. Statistical analyses were performed using SPSS 22.0 (IBM, Armonk, NY, USA). Two-sided *P*-values <0.05 were considered significant.

## Results

### General characteristics

Between January 1^st^ and October 31^st^, 2012, 288 inpatients with confirmed severe EV-A71 infection were registered in the three participating hospitals, including seven (2.43%) fatal cases. The patients were classified as severe if they experienced any neurological complications (encephalitis, acute flaccid paralysis, and/or autonomic nervous system dysregulation) and/or cardiopulmonary complications (pulmonary edema, pulmonary hemorrhage, and/or cardiorespiratory failure); otherwise, they were categorized as mild cases. There were 188 males and 100 females, with a median age of 25 months (range: 6–123, IQR: 17–36); 161 (55.9%) patients were from urban areas and 127 (44.1%) were from rural areas. Among the 288 patients, 274 (95.1%) were diagnosed with HFMD or herpangina, and the remaining 14 (4.9%) were diagnosed with neither but were positive for EV-A71 RNA and with neurological symptoms.

Seven (2.43%) patients died within 5 days after onset; these patients were aged 27.7 ± 13.2 months. The median time from onset to death was (77 h range: 32–105, IQR: 32–82). There was no difference in gender, age, weight, and demographic distribution between dead and surviving patients. Among the survivors, 258 were fully recovered, and 23 had slight neurological symptoms/signs when they were discharged from the hospital; 19 of them were followed up from 2 to 20 weeks, and none of them was found neurological sequelae.

### Neurological clinical progression

Recorded clinical manifestations and timings are listed in Table 1. Four items of prodromal symptoms were observed and seven symptoms including dizziness, palpitations, locomotors ataxia, eyes cohesion, paradoxical respiration, ptosis, and hemidrosis were not observed among our patients. Fever, rash, herpangina, startles, and myoclonic jerks/tremors were observed among more than half of the 288 cases; nearly 40% of cases showed fatigue and 25% showed vomiting. Among all severe cases, the common symptoms and signs included movement disorders (emotional movement disorder, dyskinesia, involuntary movement), autonomic dysfunction, and disturbance of consciousness.

**Table 1:** Occurrence time of signs or symptoms in 288 severe cases after EV-A71 infection (hours)

Symptoms/signs	Median (range)^a^	Mean ± SD
Prodromal symptoms (4 items)		
Fever (*n* = 284)	0 (0–96)	6.4 ± 16.7
Rash (*n* = 274)	0 (0–78)	3.3 ± 12.1
Herpangina (*n* = 145)	0 (0–78)	3.0 ± 11.9
Flu-like symptom (*n* = 7)	0	0
Vague location symptoms (6 items)		
Vomiting (*n* = 74)	30 (0–96)	35.0 ± 25.2
Persistent hyperpyrexia (*n* = 28)	36 (0–96)	40.2 ± 27.4
Hydrostomia (*n* = 18)	24 (6–54)	27.3 ± 16.8
Headache (*n* = 4)	24 (6–72)	31.5 ± 28.7
Dizziness (*n* = 0)	—	—
Palpitation (*n* = 0)	—	—
Disturbance of consciousness indicators (5 items)		
Fatigue/sleepiness (*n* = 115)	48 (0–136)	44.0 ± 26.5
Somnolence (*n* = 25)	48 (6–120)	57.4 ± 27.3
Mild coma (*n* = 7)	36 (24–96)	49.7 ± 27.0
Moderate coma (*n* = 9)	68 (27–106)	58.9 ± 25.9
Deep coma (*n* = 6)	73.5 (30–75)	59.3 ± 22.7
Autonomic dysfunction indicators (30 items)		
Poor peripheral perfusion (4 items)		
CRT extension (*n* = 27)	48 (18–114)	52.9 ± 25.4
Clammy skin (*n* = 25)	48 (18–114)	52.4 ± 26.1
Pale skin (*n* = 13)	42 (18–96)	50.9 ± 25.8
Mottled skin (*n* = 12)	53.75 (18–106)	53.5 ± 26.6
Circulatory (7 items)		
Arrhythmia (*n* = 15)	48 (12–144)	57.8 ± 38.8
Tachycardia (*n* = 12)	47.5 (24–120)	49.6 ± 26.3
Excessive tachycardia (*n* = 7)	62 (26–88)	58.1 ± 23.2
Hypertension (*n* = 7)	48 (24–83)	53.0 ± 23.6
Bradycardia (*n* = 6)	74 (48–96)	76.7 ± 18.0
Ultrahyperpyrexia (*n* = 5)	47 (26–76)	47.4 ± 21.3
Ultrahypertension (*n* = 3)	75 (72–82)	76.3 ± 5.1
Perspiration (3 items)		
Hyperhidrosis (*n* = 2)	70.5 (27–114)	70.5 ± 61.5
Adiapneustia (*n* = 2)	72 (24–120)	72.0 ± 67.9
Partial body sweating (*n* = 0)	—	—
Respiratory (9 items)		
Irregular respiratory rhythm (*n* = 19)	48 (24–120)	60.1 ± 27.1
Tachypnea (*n* = 16)	48 (6–120)	49.1 ± 27.7
Hyperventilation (*n* = 8)	48 (12–120)	57.5 ± 35.1
Ataxia respiratory (*n* = 6)	63.5 (26–78)	57.8 ± 20.9
Apneustic breathing (*n* = 3)	29 (24–99)	50.7 ± 41.9
Cheyne-stokes breathing (*n* = 1)	—	72
Cluster breathing (*n* = 1)	—	54
Slowed breathing (*n* = 1)	—	32
Paradoxical breathing (*n* = 0)	—	—
Other autonomic dysfunction indicators (5 items)		
Frequent vomiting (*n* = 3)	24 (12–64)	33.3 ± 27.2
Erythema multiforme (*n* = 2)	70.5 (27–114)	70.5 ± 61.5
Urinary retention (*n* = 2)	—	120
Central diabetes insipidus (*n* = 1)	—	82
Anuria (*n* = 1)	—	120
Failures (2 items)		
Pulmonary edema and/or hemorrhage (*n* = 23)	72 (24–120)	62.9 ± 28.6
Refractory shock (*n* = 9)	72 (30–93)	59.9 ± 23.7
Dyskinesia indicators (46 items)		
Emotional movement disorders (6 items)		
Startled (*n* = 211)	30 (0–84)	30.0 ± 23.0
Anxiety (*n* = 39)	36 (0–136)	40.1 ± 31.1
Dysphoria (*n* = 17)	42 (0–96)	44.2 ± 23.1
Fright (*n* = 14)	48 (0–72)	45.7 ± 22.1
Gibberish (*n* = 2)	33 (24–42)	33.0 ± 12.7
Mania symptoms (*n* = 1)	—	72
Limb/muscle movement disorders (14 items)		
Myoclonic jerks/tremor (*n* = 162)	36 (0–96)	33.3 ± 23.0
Dysphagia (*n* = 8)	60 (12–68)	51.3 ± 18.4
Acute lower extremity flaccid paralysis (*n* = 12)	54 (0–125)	55.6 ± 37.8
Seizures (*n* = 10)	24 (0–96)	31.7 ± 26.9
Nuchal rigidity (*n* = 8)	59.5 (24–130)	66.5 ± 37.0
Acute extremities flaccid paralysis (*n* = 5)	72 (48–120)	72.0 ± 29.4
Neck weakness (*n* = 5)	48 (24–72)	48.0 ± 24.0
Hypomyotonia (*n* = 5)	47 (0–125)	53.6 ± 48.0
Acute upper extremity flaccid paralysis (*n* = 5)	72 (47–125)	72.8 ± 31.7
Hypermyotonia (*n* = 2)	36 (24–48)	36.0 ± 17.0
Galloping tongue (*n* = 1)	—	84
Thalamus hand/foot (*n* = 1)	—	42
Body ataxia (*n* = 0)	—	—
Ptosis (*n* = 0)	—	—
Abnormal eyeball movements (8 items)		
Nystagmus (*n* = 16)	48 (18–99)	51.8 ± 28.5
Eyeball ataxia (*n* = 10)	54 (24–99)	57.6 ± 31.7
Autokinetic eyeball (*n* = 8)	45 (18–72)	43.8 ± 18.3
Fixed eyeball (*n* = 3)	72 (60–84)	72.0 ± 17.0
Gaze (*n* = 2)	30 (18–42)	30.0 ± 16.8
Ocular abduction (*n* = 1)	—	36
Turning up eyeball (*n* = 1)	—	48
Eyeball cohesion (*n* = 0)	—	—
Involuntary movements (5 Items)		
Chew (*n* = 1)	—	60
Bruxism (*n* = 1)	—	72
Blink/frown (*n* = 1)	—	30
Suck (*n* = 1)	—	48
Catch empty air/grope (*n* = 1)	—	56
Abnormal pupil movements (6 items)		
Abnormal pupillary light reflex (PLR) (*n* = 5)	72 (32–79)	65.8 ± 19.1
Mydriasis (*n* = 4)	76.5 (36–79)	67.0 ± 20.7
Myosis (*n* = 1)	—	60
Anisocoria (*n* = 1)	—	48
Dyscoria (*n* = 1)	—	26
Variable pupil (*n* = 1)	—	60
Other neural reflexes (7 items)		
Absence of pharyngeal reflex (*n* = 8)	66 (12–120)	63.9 ± 34.7
Knee hyporeflexia (*n* = 7)	54 (36–72)	55.1 ± 12.5
Ankle clonus (*n* = 4)	54 (24–64)	49 ± 18
Knee hyperreflexia (*n* = 2)	66 (48–84)	66.0 ± 25.5
Asymmetrical knee reflex (*n* = 2)	42 (12–72)	42 ± 42.4
Babinski’s sign (*n* = 2)	48	48
Absence of corneal reflex (*n* = 1)	—	120
Death (*n* = 7)	77 (32–105)	63.4 ± 29.6

^a^A duration of 0 h indicates the initial symptoms

Among the dyskinesia indicators, emotional movement disorders were the major symptoms of dyskinesia: 211 patients (73.3%) had startles, which appeared within 30.0 ± 23.0 h as the first single symptom. Anxiety occurred in 39 patients (13.5%) and occurrence time was 40.1 ± 31.1 h. Dysphoria was observed in 17 patients, 14 with fright, two with gibberish, and one with mania. Muscle strength and muscle tone disorders were the most common symptoms of limb/muscle movement disorders. There were 162 patients (56.3%) with myoclonic jerks or tremors occurring within 33.3 ± 23.0 h, including one case of galloping tongue. There were 22 patients with acute flaccid paralysis, 12 of them involving all extremities, and 10 in the upper or lower limbs. For abnormal eyeball movement indicators, nystagmus was presents in 16 patients (5.6%) and appeared within 51.8 ± 28.5 h; 10 patients showed eyeball ataxia occurring within 57.6 ± 31.7 h; eight patients showed autokinetic eyeball within 43.8 ± 18.3 h after onset. In abnormal pupil movement indicators, abnormal light reflex was found in five cases. Pharyngeal reflex was absent in eight patients (2.8%) within 63.9 ± 34.7 h.

More than half of the patients had various levels of disturbance of consciousness. Fatigue/sleepiness (patients complain of fatigue or show disinclination to talk, decreased locomotors activity, decreased mental activity level, or sleepiness compared with their usual state) was found in 115 patients (39.9%), occurring within 44.0 ± 26.5 h, most of them complaining of sleepiness and less movements; somnolence was present in 25 patients (8.7%). There were 17 patients with different levels of coma.

Autonomic dysfunctions were the most common symptoms and signs in the seven fatal cases, but were not very common in the other severe cases (all *P* < 0.05). Twenty–eight patients (9.7%) presented poor peripheral perfusion within 53.4 ± 26.1 h, including capillary refill time (CRT) extension occurring in 27 patients (9.4%) within 52.9 ± 25.4 h; 25 patients showed clammy skin, pale skin in 13, and mottled skin in 12. In the seven fatal cases, poor peripheral perfusion occurred in the early stages (Table 4).

For the circulatory system, 15 patients (5.2%) had different kinds of arrhythmia within 57.8 ± 38.8 h. There were 12 patients (4.2%) with tachycardia within 49.6 ± 26.3 h, and seven had excessive tachycardia within 58.1 ± 23.2 h; five of them died (*P* = 0.001). Refractory shock was observed in nine patients (3.1%), and occurred within 59.9 ± 23.7 h (*P* < 0.001).

For the respiratory system, irregular respiratory rhythm was observed in 19 patients (6.6%) within 60.1 ± 27.1 h, and seven of them died (*P* = 0.001). Sixteen patients presented tachypnea within 49.1 ± 27.7 h. Eight patients were with hyperventilation within 57.5 ± 35.1 h. Pulmonary edema and/or hemorrhage was observed in 23 patients (8.0%), which appeared within 62.9 ± 28.6 h; seven of them died (*P* = 0.001).

Exceped the prodromal symptoms, the 50 symptoms/signs with the highest frequency are shown in Table 2, all of which being possibly related to neurological dysfunction. There was no significant difference in the occurrence timing of diverse symptoms/signs such as between myoclonic jerks and tremor and seizures (*P* = 0.512), persistent hyperpyrexia and anxiety (*P* = 0.997), persistent hyperpyrexia and fatigue/sleepiness (*P* = 0.904), fatigue/sleepiness and fright (*P* = 0.216), fright and dysphoria (*P* = 0.567), or nystagmus and CRT extension (*P* = 0.543).

**Table 2:** Occurrence time of the top 50 symptoms/signs (exceped the prodromal symptoms) in 288 patients after EV-A71 infection and CNS involvement

Symptoms/signs	n	Median	Min	Max	Mean	SD
Startled	211	30	0	84	30.0	23.0
Myoclonic jerks/tremors	162	36	0	96	33.3	23.0
Fatigue/sleepiness	115	48	0	136	44.0	26.5
Vomiting	74	30	0	96	35.0	25.2
Anxiety	39	36	0	136	40.1	31.1
Persistent hyperpyrexia^a^	28	36	0	96	40.2	27.4
Poor peripheral perfusion^b^	28	48	18	114	53.4	26.1
CRT^c^ extension	27	48	18	114	52.9	25.4
Somnolence	25	48	6	120	57.4	27.3
Clammy skin	25	48	18	114	52.4	26.1
NPE/pneumorrhagia^d^	23	72	24	120	62.9	28.6
Abnormal respiratory rhythm	19	48	24	120	60.1	27.9
Hydrostomia	18	24	6	54	27.3	16.8
Dysphoria	17	42	0	96	44.2	23.1
Tachypnea	16	48	6	120	49.1	27.7
Nystagmus	16	48	18	99	51.8	28.5
Arrhythmias	15	48	12	144	57.8	38.8
Frights	14	48	0	72	45.7	22.1
Pale skin	13	42	18	96	50.9	25.8
Tachycardia	12	47.5	24	120	49.6	26.3
Mottled skin	12	53.75	18	106	53.5	26.6
Acute lower extremity flaccid paralysis	12	54	0	125	55.6	37.8
Seizures	10	24	0	96	31.7	26.9
Eyeball ataxia	10	54	24	99	57.6	31.7
Moderate coma	9	68	27	106	58.9	25.9
Refractory shock	9	72	30	93	59.9	23.7
Autokinetic eyeball	8	45	18	72	43.8	18.3
Dysphagia	8	60	12	68	51.3	18.4
Decreased or absent pharyngeal reflex	8	66	12	120	63.9	34.7
Nuchal rigidity	8	59.5	24	130	66.5	37.0
Hyperventilation	8	48	12	120	57.5	35.1
Hypertension	7	48	24	83	53.0	23.6
Excessive tachycardia	7	62	26	88	58.1	23.2
Mild coma	7	36	24	96	49.7	27.0
Knee hyporeflexia	7	54	36	72	55.1	12.5
Death	7	77	32	105	63.4	29.6
Severe coma	6	73.5	30	75	59.3	22.7
Ataxic respiration	6	63.5	26	78	57.8	20.9
Bradycardia	6	74	48	96	76.7	18.0
Ultrahyperpyrexia	5	47	26	76	47.4	21.3
Neck weakness	5	48	24	72	48.0	24.0
Acute upper extremity flaccid paralysis	5	72	47	125	72.8	31.7
Acute extremities flaccid paralysis	5	72	48	120	72.0	29.4
Abnormal PLR	5	72	32	79	65.8	19.1
Hypomyotonia	5	47	0	125	53.6	48.0
Headache	4	24	6	72	31.5	28.7
Mydriasis	4	76.5	36	79	67.0	20.7
Frequent vomiting	3	24	12	64	33.3	27.2
Apneustic breathing	3	29	24	99	50.7	41.9
Ultrahypertension	3	75	72	82	76.3	5.1

*SD* standard deviation

^a^continuous high fever >2 h with treatment

^b^CRT extension, pale skin, piebald skin, limb coldness

^c^capillary refill time

^d^neurogenic pulmonary edema and hemorrhage

### Common neurological symptoms/signs in non-surviving patients

Among the seven non-surviving cases (2.43%), six were from rural areas, and four were male. The median age of death was 29 months (IQR: 12–36; six cases were ≤3 years old) and the patients weighed 12.14 ± 2.12 kg (median: 13, range: 10–15). The median time from onset to death was 77 h (range: 32–105, IQR: 32–82). All dead patients had neurological symptoms/signs (all *P* < 0.05). Among the 42 symptoms/signs (Additional file 1), 39 were neurological symptoms/signs and they occurred earlier in patients who ultimately died than in survivors (all *P* < 0.05). It could be summarized that the usual progression was fever, fatigue/sleepiness, poor peripheral perfusion, abnormal respiratory rhythm, pulmonary edema and/or hemorrhage, refractory shock, and death (Table 3).

**Table 3:** Occurrence time of common symptoms/signs in seven non-surviving patients after EV-A71 infection with CNS involvement

Variables	Fever^a^	Fatigue	Abnormal respiratory rhythm	Pulmonary edema and/or hemorrhage	Refractory shock	Death
Mean (hours)	0	34.8	52.7	53.6	59.9	63.4
SD	0	21.5	27.5	25	26.5	29.6
Min	0	6.0	24.0	24.0	30.0	32.0

^a^A duration of 0 h indicates the initial symptom

Some of the symptoms occurring in fatal cases were not present in surviving patients. Using the Pearson analysis, correlations were identified between the timing of neurological symptoms/signs and death (Table 4). The duration between the occurrence of these symptoms/signs and death was 13.3 ± 11.0 h (95%CI: 3.16–23.41). Because of the rapid progression of the fatal cases, they had no chance to undergo MRI before death. The symptoms/signs presented in Table 4 were named “the fatal neurological symptoms/signs (FNS)” because when these symptoms/signs occurred, rescue became difficult and the patients died shortly after, usually within 13.3 h. The FNS included autokinetic eyeball (non-surviving, *n* = 4; total, *n* = 8, 50.0%), eyeball ataxia (4/10, 40.0%), severe coma (6/6, 100.0%), respiratory rhythm abnormality (7/19, 36.8%), decreased or absent pharyngeal reflex (5/8, 62.5%), ultrahyperpyrexia (3/5, 60.0%), excessive tachycardia (5/7, 71.4%), pulmonary edema and/or hemorrhage (7/23, 30.4%), refractory shock (7/9, 77.8%), and ataxic respiration (4/6, 66.7%) (all *P* < 0.05) (Table 4).

**Table 4:** Correlation of the occurrence time of death with other symptoms/signs in seven non-surviving patients after EV-A71 infection with CNS involvement

Symptoms/signs	N	Mean ± SD (hours)	Range	r	P*
Autokinetic eyeball	4	36.5 ± 24.2	18–72	0.96	0.04
Ataxia eyeball	4	38.3 ± 23.3	24–73	0.989	0.011
Ultrahyperpyrexia	3	43.3 ± 28.3	26–76	1.000	0.009
Pharyngeal reflex decreased or absent	5	47.0 ± 26.5	12–73	0.894	0.041
Respiratory rhythm abnormally	7	52.7 ± 27.5	24–75	0.959	0.001
NPE	7	53.6 ± 25	24–76	0.959	0.001
Severe coma	6	57.3 ± 21.5	30–75	0.946	0.004
Excessive tachycardia	5	58.2 ± 28.3	26–88	0.993	0.001
Refractory shock	7	59.9 ± 26.5	30–93	0.996	0.000
Ataxia respiration	4	62.8 ± 24.6	26–78	0.958	0.042

*dead vs. alive patients

There were two cases of persistent excessive tachycardia and hyperthermia within 8 h of death. Four patients received pediatric advanced life support (PALS) and died 3–4.4 days after illness onset; three of them experienced excessive tachycardia and hypertension recurring 5 to 23 h until death. It should be noticed that five of the surviving patients and one non-surviving patient were not in a coma before pulmonary edema. The mortality from pulmonary edema was the last cause in the FNS in our study.

In order to determine if some factors could indicate the eventual progression to the FNS, time-correlations between the occurrence of the FNS (any component) and other symptoms/signs were analyzed using the Pearson correlation analysis. CRT extension, fatigue/sleepiness, tachycardia, anxiety, hyperventilation, vomiting, persistent hyperpyrexia, myoclonic jerks/tremors, nystagmus, startles, dysphoria, and fright were time-correlated with FNS (all *P* < 0.05). This suggests that patients who developed these symptoms/signs were prone to develop the symptoms/signs of the FNS. Table 5 presents the timing of fatal symptoms/signs with other symptoms/signs among seven non-survivors after EV-A71 infection with neurological involvement. It suggested that the symptoms/signs associated with FNS appeared earlier than the occurrence of the FNS symptoms.

**Table 5:** Correlation of the timing of fatal symptoms/signs with other symptoms/signs among seven non-survivors after EV-A71 infection with neurological involvement

Variable	CRT Extension	Fatigue/Sleepiness	Anxiety	Hyperventilation	Tachycardia	Vomit	Persistent Hyperpyrexia	Nystagmus	Myoclonic jerks/tremor	Startle	Dysphoria	Fright
N	27	115	39	8	12	74	28	16	162	211	17	14
Mean (hours)	52.9	44.0	40.1	57.5	49.6	35.0	40.2	51.8	33.3	30.0	44.2	45.7
SD	25.4	26.5	31.1	31.5	26.3	25.2	27.4	28.5	23.0	23.0	23.1	22.1
Range	18–114	0–136	0–136	12–120	24–120	0–96	0–96	18–99	0–96	0–84	0–96	0–72
p												
Autokinetic eyeball	0	0.014	0.03	0.078	0	0.047	—	0.064	0.053	0.229	—	—
Ataxia eyeball	0.001	0.002	0.008	0.003	0	0.017	—	0.003	0.053	0.03	—	—
Ultrahyperpyrexia	0.002	—	—	—	0	—	0.002	—	—	—	—	—
Pharyngeal reflex decreased or absent	0.002	0.056	0.024	0.015	—	0.05	0.161	0.008	0.256	0.172	—	—
Respiratory rhythm abnormally	0	0.004	0.006	0	0	0.012	0.029	0.104	0.048	0.094	0.011	0.278
NPE	0	0	0.013	0.001	0	0.066	0.003	0.091	0.007	0.089	0.003	0.206
Severe coma	0	0.047	—	—	—	0.009	—	—	0.021	0.006	—	0.029
Excessive Tachycardia	0.001	0.034	0.024	—	—	0.454	0.061	0.033	0.127	0.412	—	—
Refractory shock	0	0.033	0.075	0.029	—	0.182	—	0.104	—	0.15	0.053	0.074
Ataxia respiratory	0	0.038	—	—	—	0.362	0.043	—	0.102	0.334	—	—
N (*P* < 0.05)	*10*	*8*	*6*	*5*	*5*	*5*	*4*	*4*	*3*	*2*	*2*	*1*

### MRI findings

**Fig. 1:**
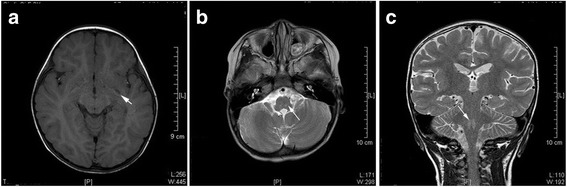
Abnormal MRI in intravalvarium. **a** Left basal ganglia (globus pallidus area) T1-WI low signal. **b**, **c** Bilateral bulbar (olive nuclear area) T2-MI high signal

**Fig. 2:**
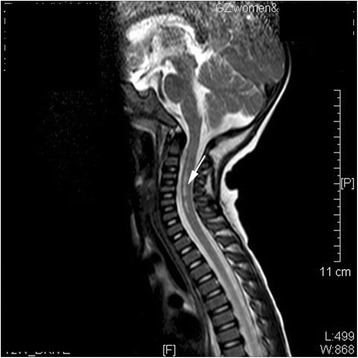
High MRI signal in C2-C7

**Fig. 3:**
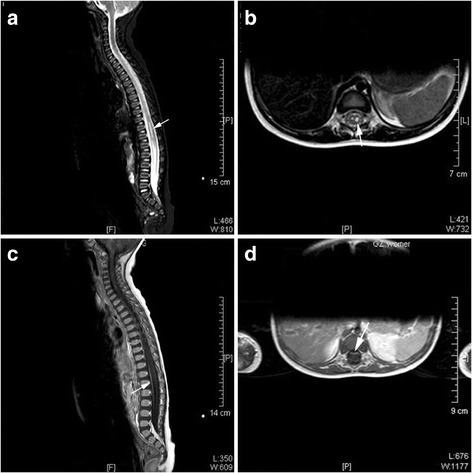
Abnormal MRI signal in the thoracic spinal cord. The spinal cord at the T10-T12 levels appeared a little swollen, with strip-liked abnormal signal in the bilateral anterior horn regions on the sagittal (**a**, **c**) and axial (**b**) views. This change was more obvious on the left side. The lesions are hyperintense on T2-WI and hypointense on T1-WI. There was no change on T1-enhancement of the lesions. Contrast-enhancement on the axial T1-WI showed strong enhancement of the ventral root (**d**)

Because of rapid death, MRI could not be performed in non-surviving patients. Among the surviving patients, 77 underwent head and spinal cord MRI examination, and MRI was abnormal in 37.7% (29/77) of patients. The affected neural structures included the thalamus, basal ganglia, midbrain and rhombencephalon, and spinal cord (Table 6). Among them, there were 65.5% (19/29) cases with pathological changes of the brain stem; 17.24% (5/29) with changes in the telencephalon/meningeal. Abnormal signals were also found in the midbrain, pon, medulla oblongata, basal ganglia, globus pallidus, and olive (Fig. 1); 10.34% (3/29) with changes in spinal cord/meninges, of which one was combined with changes in the spinal cord and/or ventral roots; and 6.90% (2/29) showed changes in the spinal nerve roots only (Figs. 2 and 3). No changes were found in the other 48 cases. Among the cases with MRI changes in the brain stem, clinicians observed 14 cases of myoclonic jerks/tremors, 14 of fatigue/sleepiness, 11 of startles, nine of persistent hyperpyrexia, seven of vomiting, seven of disturbance of consciousness, seven of abnormal respiratory rhythm, seven of NPE, five of anxiety symptoms, five of CRT extension, and five of arrhythmia, as well as some cases of hyperventilation, ataxic respiratory, eye movement disorders, abnormal pupil movements, autonomic dysfunction, and shock.

**Table 6:** Relationship between symptoms/signs and pathological MRI changes

Symptoms/signs	Negative results, but with symptoms (n)	Fatal cases (n)	Total cases with symptoms (n)	n^a^	Nerve activity may be involved	Neuron/cerebral nuclei	Lesion localization in MRI
Startled	23	4	211	20	Startle Reflex	Anterior horn motor neurons	Amygdala
					(Short-pathway) Motor nerve fibers; anterior horn of the spinal cord; amygdala, caudate nucleus; thalamus	Amygdala	Caudate nucleus
					(Long pathway) Short-pathway and frontal cortex	Caudate nucleus; thalamus	Thalamus
						Limbic system	Cortex
						Frontal Cortex	
Myoclonic jerks/tremors	26	1	162	20	Extrapyramidal System & Pyramidal System	Olive-Red Nucleus-Dentate Nucleus;	Olive nucleus
						Red Nucleus	Basal ganglia
					Motor nerve fibers in head, face and neck	Basal Ganglia	Dentate nucleus
					Rubrospinal tract	Cranial nerve nuclei	Spinal
					Brainstem spinal tract	Spinal motor neurons	Spinal nerve roots
					Extrapyramidal tract	Spinal nerve roots	
					Pyramidal tract		
Fatigue/sleepiness	25	7	115	19	Tonic stretch reflex	Spinal cord	Spinal cord
					Muscle tension	Brainstem	Brainstem
						Basal ganglia	Basal ganglia
						Cortex	Cortex
						Cerebellum	
Vomiting	11	4	74	11	Vomiting reflex	Olive nucleus	Olive nucleus
					Vagus; sympathetic	Spinal motor neurons	Spinal motor neurons
					Phrenic nerve		
					Spinal nerve		
Anxiety	3	3	39	8	Defensive reflex	Striaterminalis	Basal ganglia
					Motor nerve fibers in head, face and neck	amygdala; hypothalamus	Thalamus
						Midbrain	
						Limbic system	
CRT extension	2	7	27	7	Vascular autonomic regulation	Brainstem	Brainstem
					Sympathetic (α)	Spinal cord	Spinal cord
						Sympathetic trunk	
Somnolence	4	5	25	7	Sleep-awareness cycle	Midbrain	Brainstem
						Pons	Midbrain
						Coeruleus	Pons
						Medullary reticular formation	
Abnormal respiratory rhythm	2	7	19	7	Anterior horn motor nerve fibers	Pons	Pons
Pulmonary edema and/or hemorrhage	7	7	23	7	Sympathetic-mediated	Brainstem	Brainstem
						Spinal cord	Thoracic spinal cord Spinal nerve roots
						Sympathetic trunk	
Tachycardia	4	2	12	6	Cardiovascular autonomic regulation	Brainstem	Brainstem
					Sympathetic	Spinal cord	Spinal cord
					Vagus nerve	Sympathetic trunk	
Nystagmus	0	2	16	5	Cranial nerve III, IV, VI	Cranial nerve nuclei III, IV, VI	Midbrain
							Pons
Eyeball ataxia	1	4	10	4	Cranial nerve III, IV, VI	Cranial nerve nuclei III, IV, VI	Midbrain
							Pons
Hyperventilation	1	7	8	4	Anterior horn motor nerve fibers	Midbrain	Midbrain
Flaccid paralysis	0	1	12	4	Motor nerve fibers	Anterior horn motor neurons	Spinal cord
					Spinal nerve roots	Spinal cord	Spinal nerve roots
					Anterior horn of the spinal cord		
Fright	3	2	14	3	Defensive reflex	Amygdala; Hypothalamus	Hypothalamus
					(Short-pathway) Anterior horn motor nerve fibers; amygdala; Caudate nucleus; thalamus	Dorsal central midbrain	Midbrain
					(Long pathway) Short path and cortex	Limbic system	
Absent pharyngeal reflex	1	4	8	3	Pharyngeal reflex	Cranial nerve nuclei V, IX, X, XII	Midbrain
					Trigeminal; vagus		Pons
					Glossopharyngeal		Medulla oblongata
					Hypoglossal		

^a^Total number with pathological changes was 29 patients among the 77 survivors who underwent head and spinal cord MRI examination

Among patients with telencephalon/meningeal changes, five cases showed startles and fatigue/sleepiness, four with vomiting, four with myoclonic jerks/tremors, three with persistent hyperpyrexia, three with mental disorders, and even disturbance of consciousness and central respiratory disorders.

Among the cases with MRI changes in the brain stem, disturbance of consciousness or mental disorders were not found, but startles, myoclonic jerks/tremors, decreased muscle tension, decreased muscle strength and acute flaccid paralysis were observed.

### Autopsy findings

**Fig. 4:**
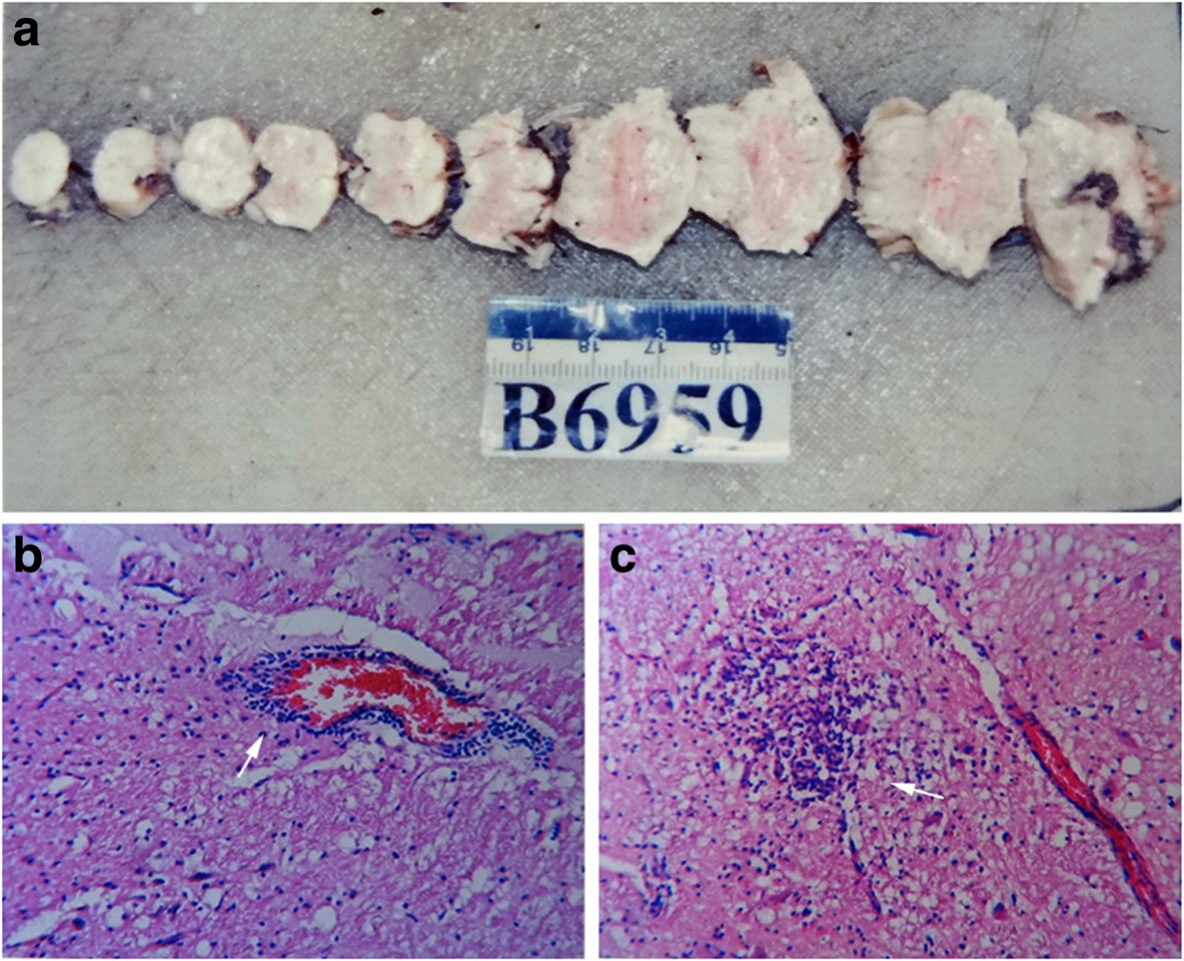
Micrographs of brain biopsy specimens obtained from patients with EV17 infection. **a** Gross specimen. Sections were stained using hematoxylin and eosin. **b** Perivascular cuffing in the brain stem (*arrow*). **c** Colloid in the brain stem (*arrow*)

**Fig. 5:**
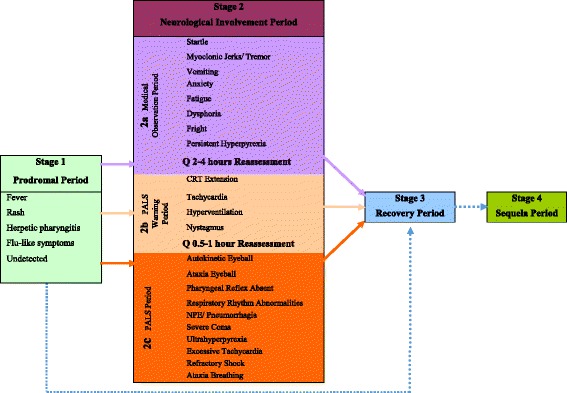
Staging of the infection progression

Two fatal cases revealed neuronal necrosis, softening, perivascular cuffing, colloid, and the neuronophagia phenomenon in the brainstem (Figs. 4 and 5). They had pulmonary hemorrhage without inflammation. Cardiac specimens showed breakage or undulatory array of some myocardial fibers without inflammation, necrosis, or hemorrhage. There were no pathological changes in other organs.

## Discussion

Among the severe cases of EV-A71 infection identified in the present study, neurological symptoms and signs occurred at each stage, which confirmed the neurotropic nature of EV-A71. Some of the symptoms such as myoclonic jerks/tremors and vomiting related to EV-A71 infection could not be related to damages in specific neural region [14–16]. However, in view of the observations, the symptoms could be generalized into two groups according to the neural structures involved: motor-control, involving the thalamus, basal ganglia, midbrain and rhombencephalon, spinal cord and ventral roots; and autonomic nerve system. The former was more frequent among surviving patients, and the latter mostly occurred in fatal cases [17]. Referring to the relevant literatures, SCARB2 is essential for neurological involvement with EV-A71 infection [18–22]. EV-A71 binds to myelin SCARB2, which induce degranulation and neuron damage [19, 20]. Nagata et al. [15, 17, 23], in a macaque model of EV-A71 infection, showed that damaged neural area included limbic system, pyramidal system, extrapyramidal, and autonomic nerves. By binding to SCARB2 receptors on myelin of these regions, EV-A71 can attack the brainstem in a few hours through reverse axonal transport [14, 24–26]. Autopsy studies showed that the brain, especially the brain stem, was most severely involved [27–30]. Neurons in areas of inflammation and tissue necrosis have been shown to be positive for EV A71 by immunohistochemistry [31]. These findings were similar to the MRI and autopsy findings of the present study, and could explain the neurological manifestations. Nevertheless, additional studies are necessary to determine whether myelin structures provide a direct neural pathway for EV-A71 invasion.

In the present study, the age distribution (median: 25 months; IQR: 17–36) was not only similar to a large study from China [2], but also to a number of previous studies [3, 4, 7]. Whether the age distribution of severe vulnerability is related to active myelination [24, 32–37], will have to be confirmed in a future study.

It has been suggested that the duration from onset to present neurological symptoms also depends on the velocity of reverse axonal transportation, the length of motor nerve and the characteristics of the virus including its virulence, mode of spread and transmission. In the present study, seven patients died within 5 days after onset with autonomic dysfunction. All of them developed subsequent irregular respiration, pulmonary edema/hemorrhage, and refractory shock. The rapid progress of fatal cases might be explained by the direct EV-A71 invasion of the medulla oblongata via the cranial nerve (CN). The autopsy showed that the cause of death was acute central nervous failure after brain stem encephalitis. Two dead patients showed neuronal necrosis, softening, perivascular cuffing, colloid, and neuronophagia phenomenon in the brainstem. The brain and cerebellum lesions were very similar. Pulmonary necrosis and inflammatory cell infiltration were not observed, suggesting that they had pulmonary hemorrhage without inflammation. Cardiac specimens showed breakage or undulatory array of some myocardial fibers without inflammation, necrosis, or hemorrhage. There were no pathological changes in other organs. Therefore, the pathologists confirmed that these patients died of acute central nervous system dysfunction caused by brainstem encephalitis.

The occurrence timing of some neurological symptoms or signs that indicate nerve lesions was not different between the survivors and non-survivors. Therefore, it may be hypothesized that the virus invasion path may go through multiple neural pathways or different cranial nerve fibers, but this will have to be confirmed. Six cases presented pulmonary edema/hemorrhage without coma, and one of them had abnormal MRI signals in the spinal cord and spinal roots without abnormal change in brain. This phenomenon might be related to the direct attack on the medulla oblongata, spinal cord or sympathetic trunk, without involving awakening centers [38]. The positive MRI findings were consistent with the clinical manifestations, including findings similar to those of poliovirus-associated poliomyelitis [39, 40] and EV-A71 infection [41]. Further studies should focus on the analysis of the pathological changes in motor nervous fibers, pyramidal, extrapyramidal tract, autonomic nerves, spinal roots, spinal cord, and each relevant motor nucleus in the brain. EV-A71 immunohistochemistry, viral load and immunohistochemistry of EV-A71 in muscles, airway, and intestinal tract should be assessed.

Data of the present study showed that when FNS occurred, patients could die in a short time. Clinicians need to pay great attention to these symptoms/signs. FNS are mainly symptoms caused by damage to the medulla oblongata, including autokinetic eyeball, eyeball ataxia, severe coma, respiratory rhythm abnormality, decreased or absent pharyngeal reflex, ultrahyperpyrexia, excessive tachycardia, pulmonary edema and/or hemorrhage, refractory shock, and ataxic respiratory.

Among the patients that underwent MRI examination in the acute phase, patients with positive MRI results showed some neurological symptoms and signs. On the other hand, in the present study, 62.3% of patients had normal MRI results, while patients with encephalomyelitis may have lesions of both the brainstem and the spinal cord. This delay could be associated with MRI time lag in acute neurologic injury or necrosis [42–44], i.e., that MRI findings may lag behind the occurrence of clinical manifestations, or that slight neurological damage does not lead to changes in imaging. DWI could be tried to improve early detection rates [45, 46]. After the initial acute illness, some lesions on follow-up MRI examinations have been shown to persist during follow-up, but some patients have no neurological sequelae, and the visualized lesions often disappear after treatment [47–50]. Nevertheless, the MRI findings may help for management and prognosis [49, 50].

The severe EV-A71 infected patients might deteriorate and even die in a short time, therefore hindering practice of neuroimaging. In addition, some uncertain factors affected the implementation of MRI in early phase, such as parent’s wishes, equipment condition, patient tolerance and checking-time selection. Primary assessments based on symptoms and signs are advantageous in these circumstances [51]. Because of rapid death, MRI could not be performed in non-surviving patients, but it could be used to determine long-term prognosis in survivors. In the presence of autokinetic eyeball, eyeball ataxia, severe coma, respiratory rhythm abnormality, decreased or absent pharyngeal reflex, ultrahyperpyrexia, excessive tachycardia, pulmonary edema and/or hemorrhage, refractory shock, and ataxic respiratory, severe damage to the medulla oblongata should be suspected, and physicians should be reminded that the patient could die very soon.

The results of the present study could be used to propose a staging system for severe EV-A71 infection. We regarded patients with FNS as Stage 2c (Fig. 4), which requires PALS. There were four indicators with time-correlation with half of the symptoms of FNS, and close to the occurring-time of FNS; this stage was named Stage 2b (PALS warning period), including CRT extension, tachycardia, hyperventilation, and nystagmus. These patients need intensive treatment, or PALS when deteriorating. This PALS warning stage is the most critical for clinical early assessment. Patients in this stage should be assessed every 30 to 60 min. Eight other indicators had time-correlation with less than half the symptoms of FNS, and far from the occurring-time of FNS; this stage was named Stage 2a (medical observation period), when startles, myoclonic jerks/tremors, vomiting, anxiety, fatigue, dysphoria, fright, or persistent hyperpyrexia appeared to imply nervous system involvement, and the patients need medical observation. The clinical progression can be directly from Stage 1 to Stage 2a, 2b, 2c or Stage 3; it can also be directly from Stage 2a, 2b or 2c to Stage 3 (recovery period). In the present study, 80.9% (233/288) of the patients directly progressed from Stage 2a to Stage 3, while 6.6% (19/288) progressed to Stage 2b before recovery. Because of the relatively sufficient preparation of advanced life support according to fully understanding the symptoms/signs, 80.6% (29/36) patients that have entered Stage 2c (36/288 cases) recovered well.

Taiwan summarized the epidemiology and clinical features of EV-A71 infection and showed that nervous system involvement was an important factor in critically ill patients [47]. China mainland also obtain similar data during the outbreak of HFMD in 2008. Based on these clinical findings and our previous clinical observations, the present study refined the neurological observations and analysis, leading us to propose a clinical staging. The Taiwanese clinical staging of EV-A71 infection in Taiwan (from 1999 to 2006) included four stages [47]. Taiwan’s Stage 1 is uncomplicated EV-A71 infection, including HFMD and herpangina. Our Stage 1 is similar to the Taiwan’s Stage 1. Taiwan’s Stage 2 is complicated EV-A71 infection with CNS involvement, and Stage 3 is complicated with cardiopulmonary failure or pulmonary edema, including Stages 3A and 3B. At present, the cardiopulmonary failure or pulmonary edema of EV-A71 involvement has been considered neurogenic factors [2, 30, 52]. Our Stage 2 (neurological involvement period), includes Taiwan’s Stages 2 and 3, focusing on neurological assessment. When developing to Stage 2, patients can present different clinical manifestations caused by different neurologic conditions. The Stages 2a, 2b, and 2c may not occur sequentially, but separately, sequentially or simultaneously. The most serious neurologic involvement is in Stage 2c, in which the lesions are located in the medulla oblongata. Our Stages 3 (recovery) and 4 (sequelae) are equivalent to Taiwan’s Stage 4. Our staging system are more similar to the neurotropic characteristics of EV-A71. Depending on the severity of the neurological involvement, our clinical staging delineates different intervals for reassessment and intervention. Clinicians can look for early warning indicators to identify critical cases that are indicators for Stage 2b.

### Limitations

This study has some limitations. First, the cases were only from coastal cities in the Guangdong Province, and the generalisability might be challengeable. In addition, the sample size was small, precluding any reliable calculation of sensitivity and specificity. Secondly, we only interpreted the observation and analysis of the clinical facts. The present facts supported that neurological symptoms were valid for early assessment. Thirdly, due to ethical reasons, autopsy only confirmed the major cause of death, and we could not perform a comprehensive and exploratory autopsy; thus, brainstem lesions could not be explained as an independent reason for fatality. There could be a slight difference between the time of symptom onset and the time of symptom observation, but this difference was of maximum 15–30 min.

## Conclusions

Severely affected patients may die from EV-A71 infection. Close attention and early assessment of certain symptoms/signs is important for severe infection administration. Symptomatic treatment is warranted in all patients.

## Additional files

Additional file 1: Explanations of the observed indicators. Indicators without descriptions were standard indicators assessed according to the *Diagnostics* textbook.

## Abbreviations

CN: Cranial nerve
CNS: Central nervous system
CRT: Capillary refill time
EV-A71: Enterovirus 71
FNS: Fatal neurological symptoms/signs
HFMD: Foot and mouth disease
