# Supplementary material for: Clinical manifestations of severe enterovirus 71 infection and early assessment in a Southern China population
Source: BMC Infect Dis. 2017 Feb 17;17:153. doi: 10.1186/s12879-017-2228-9 (PMC5316173; doi:10.1186/s12879-017-2228-9)
Supplement: Additional file 1: — Explanations of the observed indicators. Indicators without descriptions were standard indicators assessed according to the Diagnostics textbook. [file 12879_2017_2228_MOESM1_ESM.doc]

**Additional file 1.** Explanations of the observed indicators. Indicators without descriptions were standard indicators assessed according to the *Diagnostics* textbook.

**Prodromal** **indicators (4 items):** fever, rash, herpangina, and flu-like symptoms.

**Vague location indicators (6 items)**

Vomiting——vomiting per hour less than 2 times.

Persistent fever——after using conventional physical and drug cooling methods, the temperature remained over 39°C for more than 2 hours.

Hydrostomia

Headache—— more than 3-year-old children complain from forehead, or roof, or temporal, or occipital pain.

Dizziness—— more than 3-year-old children complain from sense of insecurity and instability with standing and walking difficulties, or feeling that the surrounding environment is floating or rotating, or head swinging, or feeling faint, or with transient loss of consciousness.

Palpitation[^3^](#_ENREF_3)—— more than 3-year-old children complain from palpitation, or heartbeat discomfort, or strong heartbeat, or shock, or arrhythmias.

**Dyskinesia (46 items)**

Emotional Movement Disorders (6 items)

Startle—— paroxysmal rapid contraction and buckling of facial and limb muscles, may accompany expression muscle movement, such as frowning and winking.

Anxiety—— patients is nervous, restless, irritable, or excited.

Dysphoria—— patients show boredom, impatience, irritability, restlessness, and hand and foot restlessness.

Fright[^6^](#_ENREF_6)—— patients suddenly tense, eyes widened, expression of fear, fast breathing, muscle tension, with dodging reflex.

Anxiety symptoms—— patients show high emotion, hypermetamorphosis, language increase, enhanced initiative, action more, psychomotor excitement.

Gibberish—— including delirium and paraphasia with or without disturbance of consciousness.

Limbs/Muscles Movement Disorder (14 items)

Myoclonic tremors —— short-speed, rigid, involuntary, low-level (with consciousness), or swing, or single-limb shaking movements, or upper and lower extremities simultaneously movement.

Dysphagia—— accumulation of saliva in the mouth, unsuccessful food swallowing, may even lead to aspiration.

Seizures—— sudden, rapid, usually over performance of stereotyped clonic movement.

Limb myasthenia—— limb muscle strength decline.

Neck stiffness—— neck cannot bend.

Neck weakness—— more than 3-month-old patients cannot rise head persistently or vertically.

Hypermyotonia

Hypomyotonia

Upper extremity weakness—— declined muscle strength of upper extremity.

Lower extremity weakness—— declined muscle strength of lower extremity.

Galloping tongue—— episodic, rhythmic involuntary movements of the tongue, starting from shrinkage of the partial midline lingualis in back of the tongue.

Thalamus hand/foot——wrist and metacarpophalangeal joint flexion, or ankle and metatarsal metacarpal joint flexion, and finger/interphalangeal joint hyperextension.

Body ataxia—— under normal circumstances of muscle strength, amplitude or coordination of limb voluntary movement disorder, and patients cannot maintain body posture and balance.

Ptosis

Abnormal Eyeball Movement (8 items)

Nystagmus—— paroxysmal nystagmus, bilateral eyeballs synchronous quickly two-phase horizontal/vertical vibration according to a certain amplitude.

Autokinetic Eyeball—— synchronous movement of bilateral eyeballs without specific rate and amplitude.

Ataxic Eyeball—— asynchronous and irregular movement of bilateral eyeballs

Ocular Abduction.

Gaze

Turning Up Eyeball

Eyeball Fixed

Eyeball Cohesion

Involuntary Movement (5 Items)

Chewing

Odontoprisis

Blinking / Frowning

Sucking

Catch Empty Air/ Grope

Abnormal Pupil Movement (6 items)

Abnormal pupillary light reflex (PLR)

Myosis—— the diameter of pupil is <2 mm.

Mydriasis—— the diameter of pupil is >5 mm.

Anisocoria—— the diameter of bilateral pupils are unequal.

Dyscoria—— the shape of bilateral pupils are not the same.

Pupil Variable—— asymmetry of bilateral pupils change .

Other Neural Reflex (7 items)

Absence of Pharyngeal Reflex

Absence of Corneal Reflex

Pathological Reflex

Ankle Clonus

Knee Hyperreflexia

Knee Hyporeflexia

Asymmetrical Knee Reflex

**Autonomic dysfunction (30 items)**

Poor Peripheral Perfusion[^11^](#_ENREF_11) (4 items)

CRT Extension—— CRT > 3sec.

Clammy Skin

Ochrodermia

Mottled Skin

Circulatory (7 items)

Tachycardia—— heart rate >1 SD.

Ultrahyperpyrexia—— Temperature >41°C.[^12-14^](#_ENREF_12)

Excessive tachycardia —— heart rate >220 bpm.[^15^](#_ENREF_15)

Ultrahypertension—— blood pressure >2 SD, or >(99^th^ percentile + 5mmHg).[^16^](#_ENREF_16)

Hypertension—— blood pressure >1 SD, or 95^th^ - (99^th^ percentile + 5mmHg).[^16^](#_ENREF_16)

Arrhythmia—— any arrhythmia except tachycardia and bradycardia.

Bradycardia—— heart rate slower than normal.

Perspiration (3 items)

Hyperhidrosis

Adiapneustia

Partial body sweating

Respiratory (9 items)

Tachypnea—— respiration is regular but faster than normal.

Hyperventilation—— tachypnea with arterial blood gas PaCO2 <35 mmHg.

Irregular respiratory rhythm

Ataxia respiratory—— inspiratory with completely irregular amplitude and length, mixed apnea.

Cheyne-Stokes breathing

Apneustic breathing—— long inhalations, then pause for a few seconds, and then exhalation

Cluster breathing[^17^](#_ENREF_17)—— pattern of periodic breathing, an alternation between respiratory pause and inspiration with wax and wane in amplitude until the next apnea.

Slowed Breathing

Paradixic breathing—— the opposite movement of thoracic and abdominal. breathing

Other autonomic dysfunction indicators (4 items)

Frequent vomiting—— vomiting >2 times per hour.

Erythema multiforme—— the sudden appearance of erythema on the skin, spreading and fading rapidly.

Central diabetes insipidus[^18^](#_ENREF_18)—— urine volume is >200 ml/h, or more than 6 ml/kg/h.

Anuria—— urine volume is <100 ml/24 h, or <0.5 ml/h, or complete anuria in 12 h.

Failure (2 items)

Refractory shock—— serious poor peripheral perfusion, thready and rapid pulse, blood pressure drops, continued active treatment of shock is invalid.

Pulmonary edema and/or hemorrhage—— suddenly appearance of pulmonary rales when reassessment, or spumous phlegmy and/or bloody exudate occur in endotracheal tube, oral, or nasal cavity.

**Disturbance of consciousness (5 items):**

Fatigue/sleepiness—— Patients complain of fatigue, or present disinclination to talk and less locomotors activity compared with the previous, or mental activity level is decreased, or sleepiness.

Somnolence

Mild coma

Moderate coma

Deep coma

**References**

1. Drachman DA, Hart CW. An approach to the dizzy patient. Neurology 1972;22:323-34.

2. Baloh RW. Approach to the evaluation of the dizzy patient. Otolaryngology--head and neck surgery : official journal of American Academy of Otolaryngology-Head and Neck Surgery 1995;112:3-7.

3. Fricker P. Anterior knee pain [published errartum appears in Aust Fam Physician 1989 Mar;18(3):195]. Australian family physician 1988;17:1055-6.

4. Brown P. Neurophysiology of the startle syndrome and hyperekplexia. Advances in neurology 2002;89:153-9.

5. Carlsen AN, Chua R, Inglis JT, Sanderson DJ, Franks IM. Differential effects of startle on reaction time for finger and arm movements. Journal of neurophysiology 2009;101:306-14.

6. Gorman JM, Askanazi J, Liebowitz MR, et al. Response to hyperventilation in a group of patients with panic disorder. The American journal of psychiatry 1984;141:857-61.

7. Baquis GD, Pessin MS, Scott RM. Limb shaking--a carotid TIA. Stroke; a journal of cerebral circulation 1985;16:444-8.

8. JB. G. Loss of Control.Clinical Methods: The History, Physical, and Laboratory Examinations. 3rd. ed. Boston: Butterworths1990.

9. DF. B. Aphasia, alexia, and agraphia. : New York: Churchill-Livingstone; 1979.

10. Keane JR. Galloping tongue: post-traumatic, episodic, rhythmic movements. Neurology 1984;34:251-2.

11. Lima A, Bakker J. Noninvasive monitoring of peripheral perfusion. Intensive care medicine 2005;31:1316-26.

12. Trautner BW, Caviness AC, Gerlacher GR, Demmler G, Macias CG. Prospective evaluation of the risk of serious bacterial infection in children who present to the emergency department with hyperpyrexia (temperature of 106 degrees F or higher). Pediatrics 2006;118:34-40.

13. Saunders P. Manson's Tropical Diseases: Expert Consult. 2008:1229.

14. Zhao CL, Mao HJ, Xia F, Chen XY. Fourteen cases of neonatal ultrahyperpyrexia syndrome induced by improper care. Advances in therapy 2007;24:101-5.

15. Jackson AS. Estimating maximum heart rate from age: is it a linear relationship? Medicine and science in sports and exercise 2007;39:821.

16. Badeli H, Sajedi SA. Childhood hypertension: new concepts, definitions, and diagnostic evaluations. Iranian journal of kidney diseases 2008;2:123-6.

17. Weintraub Z, Cates D, Kwiatkowski K, Al-Hathlol K, Hussain A, Rigatto H. The morphology of periodic breathing in infants and adults. Respiration physiology 2001;127:173-84.

18. Lugo N, Silver P, Nimkoff L, Caronia C, Sagy M. Diagnosis and management algorithm of acute onset of central diabetes insipidus in critically ill children. Journal of pediatric endocrinology & metabolism : JPEM 1997;10:633-9.
